# Work and politics in the era of liquid modernity: meanings attributed by female nurses^*^

**DOI:** 10.1590/1980-220X-REEUSP-2025-0175en

**Published:** 2025-12-12

**Authors:** Midian Oliveira Dias, Juliana Amaral Prata, Ana Beatriz Azevedo Queiroz, Carolina Cabral Pereira da Costa, Samira Silva Santos Soares, Luanny Regina de Oliveira Santos, Luana dos Santos Cunha de Lima, Norma Valéria Dantas de Souza

**Affiliations:** 1 Universidade do Estado do Rio de Janeiro, Faculdade de Enfermagem, Departamento de Enfermagem Materno-Infantil, Rio de Janeiro, RJ, Brazil.; 2 Universidade Federal do Rio de Janeiro, Escola de Enfermagem Anna Nery, Departamento de Enfermagem Materno-Infantil, Rio de Janeiro, RJ, Brazil.; 3 Universidade do Estado do Rio de Janeiro, Faculdade de Enfermagem, Departamento de Enfermagem Médico-Cirúrgico, Rio de Janeiro, RJ, Brazil.; 4 Universidade Estadual de Santa Cruz, Departamento de Ciências da Saúde, Ilhéus, BA, Brazil.; 5 Universidade Estácio de Sá, Rio de Janeiro, RJ, Brazil.; 6 Centro Federal de Educação Tecnológica Celso Suckow da Fonseca, Rio de Janeiro, RJ, Brazil.

**Keywords:** Nursing, Work, Politics, Work Engagement, Capitalism.

## Abstract

**Objective:**

To understand the meanings attributed by female nurses to work and politics in liquid modernity.

**Method:**

This qualitative study involved 46 graduate faculty students of a public state university in the city of Rio de Janeiro. Data were collected from April to November 2021 through semi-structured individual interviews and submitted to IRaMuTeQ^®^.

**Results:**

Similarity analysis was structured around two central axes (“work” and “politics”), surrounded by peripheral words. The first emerged as a means of subsistence. The second is seen as yet another duty amid the intensification of work, which distances female nurses from political activity, even though they recognize its importance in achieving collective benefits. Regarding professional associations, they view them as a financial investment with little or no concrete return.

**Conclusion:**

Concerning liquid modernity, there is an emptying of meanings associated with work concomitant with the devaluation of class entities, culminating in the removal of female nurses from political action.

## INTRODUCTION

Work can be defined as any activity that requires the expenditure of physical and/or mental energy, directly or indirectly, for the purpose of producing goods and/or services, permeated with meanings and values according to the cultural and historical contexts that permeate them. Furthermore, it has the capacity to contribute to the construction of personal and collective subjectivities and identities, as well as offering guarantees of subsistence, usefulness, security, belonging to a group, social status, among others^(1,2,3)^.

However, in contemporary times, there has been a paradigm shift in the way people are and exist in the world, with the consequent transformation of humankind’s relationship with work, a phenomenon described by sociologist Zygmunt Bauman as liquid modernity. With the incorporation of the neoliberal capitalist political-economic-ideological model, work began to take on a fluid, flexible, and precarious dimension, losing its centrality in human life, and consumption began to occupy this place^(4,5)^.

In metaphorical allusion to the liquid state of matter, which is easily molded and adaptable to different environmental conditions, this complex social rearrangement is currently unstable and with undefined dimensions^(4,5,6)^. Furthermore, there is superficiality and flexibility in social relations, acceleration of the pace of life, impersonality, individualism, consumerism, and a propensity for rapid changes, according to political and social nuances^(4,5,6)^.

This context goes against human nature, which is permeated by essentially political and social elements, which have been deconstructed by liquid modernity. In the social sphere, consumption promotes instrumentalization of social relations, reducing them to consumer relations. Thus, there is objectification of the other, consuming their usefulness and discarding them when they can no longer satisfy individual desires^(4,5,6)^.

Considering politics as a means of resolving conflicts and organising society, it is worth noting that liquid modernity has reshaped human existence in various ways, emphasising individualism over the collective. This has direct repercussions for political participation, including in the workplace^(7,8)^.

Given this understanding, and applying it to the specific world of nursing, there is a consensus in the scientific literature that reveals nursing’s avoidance and disinterest in discussing politics in the workplace. Therefore, it is believed that liquid modernity has significantly altered nursing’s and society’s relationships with politics^(4,5,6)^.

Bauman classifies individualism, short-termism, and consumerism as reasons for human distancing from politics. Self-centered people are unwilling to dedicate energy and time to finding solutions to collective problems, which require courage and dedication. The results are generally long-term and do not satisfy individual desires quickly^(4,5,6)^.

Given that this context leads to a loss of meaning in work due to the emptying of meanings associated with labor and the dilution of class feelings, it is necessary to understand the nuances of these transformations, in order to encourage reflections among the professional collective regarding the development of coping and overcoming strategies^(3,4)^.

In this context, the importance of this study emerges, especially considering that, in Brazil, female nurses’ work reality can contribute to political apathy, since these workers live with low wages, an accumulation of contracts and exhausting working hours developed amid polyvalence, intensification and inadequacies in working conditions^(9)^. Given these findings, the present study aimed to understand the meanings attributed by female nurses to work and politics in the era of liquid modernity.

## METHOD

### Study Design

This qualitative study followed the criteria established in the Consolidated criteria for Reporting Qualitative research^(10)^.

### Setting, Population and Selection Criteria

The study setting was a nursing school affiliated with a public state university located in the city of Rio de Janeiro, Brazil. Study participants were 46 nurses enrolled in the institution’s *lato sensu* and *stricto sensu* graduate programs. The total number of students enrolled during the data collection period was 203, of which 157 were in lato graduate programs and 46 in stricto sensu graduate programs.

Participants regularly enrolled in graduate programs during the data collection period and with at least one year of professional nursing experience, thus ensuring an understanding of the nursing workforce specificities, were included. Professionals currently working in fields not directly related to nursing and nursing residents were excluded, as this type of training requires exclusive dedication, precluding simultaneous entry into the job market.

### Data Collection

Participant recruitment began with a survey of students enrolled in *lato sensu* and *stricto sensu* graduate programs conducted by their respective academic offices. The survey was then publicized through a digital form, created and hosted on a free website, which outlined the study’s objectives and an invitation to participate. Interested participants provided contact information and indicated their intention to participate.

As an additional strategy, the author responsible for data collection also approached students in remote, technologically mediated classes, given the restrictions on in-person attendance during the COVID-19 pandemic. On these occasions, course coordinators provided a few minutes to present the study’s objectives and its relevance to nursing, followed by an invitation to participate in the research.

Ultimately, the recruitment process included 46 nurses, of whom 34 were recruited through the application form and 12 through oral interviews. It is noteworthy that there were no refusals to participate, but there were seven losses due to the poor audio quality of interview recordings.

Data were collected from April to November 2021 through individual interviews, which followed a semi-structured script divided into two parts. The first part contained ten closed-ended questions to briefly characterize participants (sex, gender identity, age, course of study, marital status, number of children, family breadwinner responsibility, type and number of employment relationships, and association between employment and other paid activities). The second part contained the following trigger question: as a female nurse, what is your perception of the relationship between politics and work?

The interviews were conducted by one of the authors via voice telephone, in the presence of the researcher and the participant. They lasted an average of 30 minutes, were recorded in MP4 audio with prior authorization, and were subsequently transcribed. It should be noted that the interviews were conducted by one of the authors, a female nurse, professor, and doctoral student in nursing at the time of data collection, with experience in producing qualitative data. She also tested the instrument with two doctoral students, who were not included in the study.

Data collection was completed when the transcribed material did not present new ideas, thus understanding that inductive thematic saturation had been reached^(11)^, which was identified in the forty-third interview and confirmed with three more. Furthermore, all participants received the transcript of their interview to confirm the information, but there was no response from them with requests for changes.

### Data Analysis and Processing

For data analysis, the *Interface de R pourles Analyses Multidimensionsionnelles de Textes et de Questionnaires* (IRaMuTeQ^®^) software was used, which is a free program anchored in the R software (free package for performing statistical analyses), enabling different processing and statistical analyses of texts.

For clarity, the texts originating from the set of interviews are called the *corpus* of analysis. Each individual interview is called a “text”, and the text excerpts (statements, word environments) are called “text segments” (TSs)^(12,13)^. Thus, the 46 interviews were transcribed and grouped into a single electronic text document, with 225 pages, and the research *corpus* had 46 texts and 2,554 STs, of which 2,362 were used, representing a satisfactory use of the material with a retention of 92.48%.

For data teratment, we opted for similarity analysis, which groups words and organizes them graphically according to their frequency. This technique is based on graph theory and allows us to observe occurrences and co-occurrences between lexicons, as well as indicating connectivities between them, contributing to the recognition of content structure and thematic cores that help address the study objective^(12,13,14)^.

Furthermore, the theoretical framework proposed by Bauman^(4)^ on liquid modernity and scientific productions on nursing work were used.

### Ethical Aspects

The research was approved by the *Universidade do Estado do Rio de Janeiro* Research Ethics Committee, under Opinion 4.145.807 and Certificate of Presentation of Ethical Consideration 32251320.0.0000.5282 on July 9, 2020. The study met the ethical aspects of research involving human subjects. Participants signed the Informed Consent Form, and their identities were protected through coding, such as the use of the letter “N” (for nurse), followed by a number corresponding to the order in which the interviews were conducted.

## RESULTS

Forty-six nurses, all graduate students, participated in this study. Thirty-four were pursuing a *lato sensu* graduate degree, six were pursuing a master’s degree, and six were pursuing a doctoral degree. The majority identified as female (40), six as male, and all participants identified as cisgender, with ages ranging from 30 to 35 years. Based on this finding, it was decided to classify participants as female, understanding that nursing is a predominantly female profession and the largest number of participants are women.

In relation to marital status, 17 were single; 18 were married; three reported being divorced; four were in a stable relationship; and four did not respond. Furthermore, 25 had no children and 21 had children. Concerning income, 13 participants were not responsible for supporting their family, while 24 had this responsibility and nine responded that they perhaps, or sometimes, were responsible for providing financially for their home/family.

Regarding employment, 29 reported having a formal job, among which: seven associated this employment with an informal paid activity; eight had two formal employment relationships, and two associated the double employment relationship with an informal paid activity; four had three or more formal employment relationships, of which two combined this condition with an informal paid activity; three did not respond; and two had recently left their formal employment relationship.

**Figure 1:**
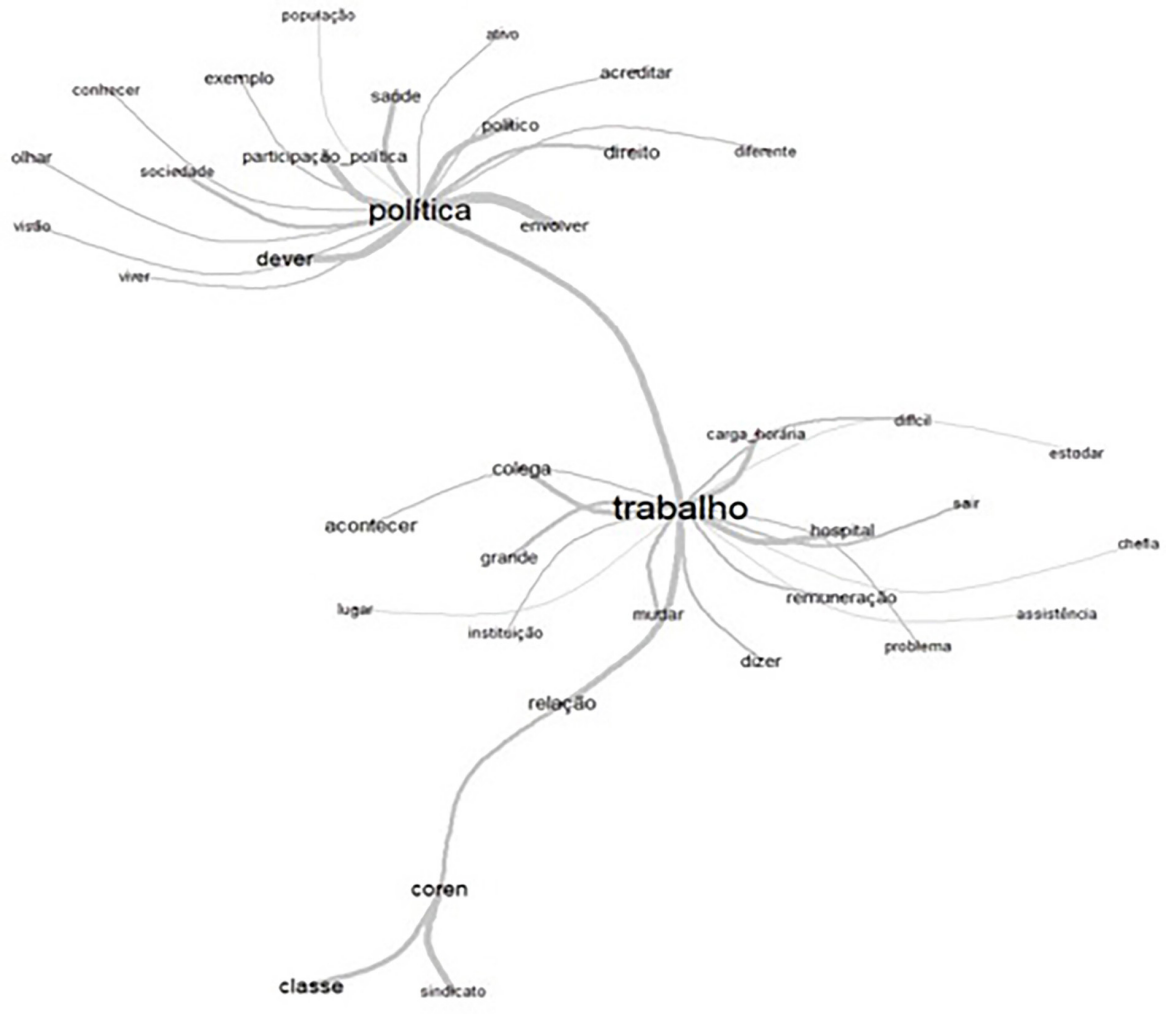
Analysis of similarities and relationships between the words “politics” and “work”

Legend: work, happen, big, change, place, institution, relationship, say, coren, class, union, remuneration, hospital, problem, assistace, head, leave, co-worker, working_hours, difficult, study, politics, involve, duty, live, right, different, believe, political, health, active, political_participation, population, example, know, society, look, view. Source: IRaMuTeQ^®^ data. Rio de Janeiro, Brazil, 2025.

In similarity analysis, the software generated Figure 1, which presents two central axes (“politics” and “work”), surrounded by peripheral words.

To elucidate the representations contained in the similarity tree, some TSs describe participants’ understanding of politics in the workplace as essential to achieving benefits. However, it is noted that they shy away from related topics, as they view it as just another workplace task.

*Politics is present in our daily work in healthcare. Nurses often end up avoiding this responsibility, as if political participation were just another burden they have to carry. Yet another duty!* (N25)

*I believe that to achieve benefits at work, we must engage in politics. Politics is present in all nursing relationships, but even knowing this, we don’t get involved in politics because we’re already overwhelmed with duties.* (N12)

*To improve my work, I engage with politics. When problems arise, I become more politically active, but in my day-to-day life, as a female nurse, I don’t get involved in politics because I already have many responsibilities.* (N08)

Furthermore, as a meaning attributed to work, TSs reveal that female nurses see work as a means of ensuring subsistence, through obtaining an wage.

*We work and struggle to survive. Paying the bills and having money is the priority. It’s the purpose of work! Our workload is already excessive! We don’t want to get involved in politics.* (N41)

*I love nursing. I love providing patient care. Even so, I work for the money, for the pay… we work for love and for money.* (N03)

*I love my profession, but love doesn’t pay the bills. We need the paycheck! The work is hard, and relationships with patients, co-workers, and management are difficult. I stay at the hospital because I need the paycheck, the remuneration to survive.* (N30)

Furthermore, some TSs demarcate the distance between workers and their entities representing the nursing category, since they consider payments to these organizations as a burden without the desired return.

*The class is abandoned, working outside of ideal conditions because we need money to survive. We lack representation. To join a union, you have to pay! The fees charged end up weighing on the budget and, furthermore, have low returns and alienate the category. This discourages.* (N12)

*I don’t see it happening! I haven’t seen the return I wanted from the union. I paid for years and, to this day, I haven’t seen any benefits. Nothing has changed. The unions are very distant from the workers. They don’t understand our problems or what we need.* (N08)

*I see many co-workers saying that COREN and the union aren’t doing anything. They just want annual payments and do nothing. That’s the rhetoric of our class.* (N18)

## DISCUSSION

In similarity analysis, the size of the words and the thickness of the lines that unite them represent how important the terms are for understanding the phenomenon studied^(15,16)^. In this regard, from the perspective of two semantic axes, it became clear that, in the first, “politics” is a central word surrounded peripherally by “political participation”, “duty”, “right”, “involve”, “society”, “health”, and other words. In the second, the lexicon “work” emerged as central and is related to the following peripheral terms: “working hours”, “remuneration”, “happen”, “coren”, “union”, “class”, among others.

From this analytical perspective, the central lexicons “politics” and “work” are the words with the thickest connecting lines, indicating greater co-occurrence (the appearance of these words together), as when female nurses report that politics is present in their daily nursing work. At the same time, there is a distance between these central words, revealing that politics is not yet an element that makes up nursing’s daily intentional actions, given that TSs demonstrate attitudes of avoidance and distancing themselves from political issues, viewing it as just another task in a scenario of overload and long work hours.

Furthermore, TSs lead to the understanding that labor is merely a means of subsistence in liquid modernity. Considering the meanings of work described by Bauman, two opposing realities are confronted. The first, called solid modernity, was characterized by a social order arising from the alliance between nation-states and science. In this scenario, workers had low mobility and low educational attainment, but their relationships with work were long-lasting and formal, and it was common to enter the labor market and retire with the same employment or role. Furthermore, work provided financial subsistence, adding social status, security, satisfaction, interpersonal relationships, and other elements essential to human beings and identity formation^(4,5,6,17,18)^.

The second reality, identified among participants in this study, emerged from changes resulting from crises that transformed the social, political, and economic landscape, such as nuclear tragedies, concentration camps, and the Holocaust, destroying the structures of solid modernity. As a result, liquid modernity was inaugurated as a logic that demarcates differences and disseminates ideals of individualization, freedom, sociability elimination, and dependence, thus opposing the paradigm of solid modernity that preceded it^(4,5,6,18)^.

This new modernity arose from the ideas of fluidity, lightness, precariousness, uncertainty, and inconstancy, bringing with it notions of practicality, adaptability, and superficiality of relationships, in addition to the acceleration of the pace of life and the exacerbation of freedom, individualism, consumerism, and flexibility. Concomitantly, there was the expansion of neoliberal capitalism and the weakening of the relationship between capital, labor, and the State, with the aim of favoring the autonomous, globalized, and free reproduction of capital. Consequently, the State became minimal, and labor flexible and precarious^(4,5,6,18)^.

In the world of work, these issues translate into a search for specialized and multifunctional workers with precarious and unstable employment relationships. Remaining in the same job or role for long periods is difficult, and when it occurs, it is frowned upon, as it reflects stagnation and lack of market progression. As a result, informal employment expands, and professional identity becomes fleeting, constantly reconstructed based on consumer-related stimuli^(4,5,6,19)^.

Considering that a person is what they consume or possess, and that consumption has come to occupy the place previously filled by work and interpersonal relationships, it follows that, in liquid modernity, individuals’ identity and subjectivities assume a fluid character^(16,17,18,19)^. Hence, it is understandable why the meaning of work for the female nurses interviewed is focused on monetary gains, which guarantee subsistence and consumption so that engaging with political issues for the sake of collective gains is not a priority. Consequently, the loss of meaning in work is aligned with the emptying of meanings due to high modern liquid demands and consumption^(19)^.

In the nursing field, this grim work environment is aggravated by nursing sociodemographic characteristics, in addition to issues of gender, historical origin, and cultural heritage of nursing, which coexist with overloaded work shifts, long working hours, low wages, and social and professional devaluation^(4,5,6)^. In contemporary times, this situation leads female nurses to political apathy, as evidenced in this research, through subjectivities related to work, given the prioritization of consumption, which can trigger the fluidity of these workers’ identities.

Corroborating this, participants’ choice to distance themselves from spaces of political discussion and struggle stands out, despite recognizing the importance of this involvement in achieving improvements in working conditions. In fact, this result reveals individualism, where personal aspirations take precedence over collective ones, and immediacy, which weakens class consciousness as elements naturalized by liquid modernity and present in all spheres of life, including work^(4,5,6, 16,17,18,19)^.

Considering the end of the second core meaning presented in the similarity tree, it is noted that the lexicon “coren” is close to “class” and “union”, indicating a correlation between these entities representing nursing and category. These words belong to the semantic spectrum of “work”, but are peripherally distant, presenting a weak connection with the central term. Thus, the distance between these lexicons indicates that there is no significant co-occurrence.

Similarly, TSs reveal that, in female nurses’ view, nursing representative entities fail to address the true needs of the category because they lack close relationships with the workers who comprise it. Thus, they view the fees charged by these organizations as a barrier to engagement, and that these expenses impact the budget. Conversely, there is a perception that membership, registration, and association yield little or no return in terms of collective benefits to professionals arising from political struggles. Thus, a cycle of alienation and a feeling of lack of representation is formed, which is accompanied by the weakening of the professional association and the consequent increase in distance.

From another perspective, it is considered that nursing representative entities represent the public sphere, which is not valued in liquid modernity, given that people are increasingly driven to remain occupied and preoccupied with their own private spheres. Thus, through the spread of individualism, the citizen self is replaced by the individual self so that, when the public sphere is occupied solely by the individual self, the collective objective ends up being diverted to an individual purpose. Thus, collective thoughts and actions cease to exist, and initiatives to publicize private life occur, leading to group alienation^(4,5,6, 19, 20)^.

In this context, there is the superficiality of relationships with peers, which prevents the construction of deep and empathetic interactions, and the centrality of consumption, as an essential component of a person’s identity, which, by reducing the notion of citizenship, places the “consumer being” above the “human being”. Furthermore, the lack of genuine social relationships and the exchange between public and private life, associated with egocentrism and insensitivity, driven by liquid modernity^(4,5,6, 19, 20)^, form an overview that distances nurses from the political sphere and, consequently, from participation in this space and the possibility of establishing a close and true relationship with the entities representing nursing.

Furthermore, it is important for professionals to understand the distinct legal competencies of each of these organizations so that they can seek information and assistance through communication channels appropriate to the nature of their demands^(21)^. To this end, nursing workers must break free from the alienation imposed by work in the era of liquid modernity and engage with their representative entities through membership, participation in scientific, cultural, social, and political assemblies and events, as well as by drafting opinions and sharing individual views on issues published in the media. Such strategies enable the exercise of social control, bring professionals closer to their peers, and strengthen nursing’s own institutions.

These issues are crucial because, today, many labor struggles culminate in legal disputes, which are met with bureaucratic and slow judicial systems. Furthermore, these processes are anchored in legislation that favors capital over workers. This is the case, for instance, with the labor and social security reforms of 2017 and 2019, which, despite promoting the idea of expanding employment and making working hours more flexible, in reality, amounted to movements that stripped working-class rights^(21,22,23)^.

In this context, it is urgent that nursing professionals and entities representing nursing organize themselves as a collective so that their struggles can conquer spaces of power and powerful decision-making to drive concrete changes in female nurses’ work reality amid the power relations of politics, the economy, and work in liquid modernity.

The study’s limitation lies in its scope, as it was conducted at only one higher education institution in the state of Rio de Janeiro, which has cultural, political, and health-related specificities that are not necessarily present in other Brazilian states. Therefore, the results may not be generalizable, but they align with findings from international research that show nursing’s low involvement in policy-related issues^(24,25)^.

Furthermore, this study provides support for critical reflections on the repercussions of liquid modernity on the labor market, nursing as a professional collective, and individual attitudes toward collective struggles in the political arena. It is noteworthy that this problematization must occur within collective work spaces, but is not limited to workers. It is urgent that this debate permeate teaching and learning processes from undergraduate to graduate levels to provide training contextualized with work and, consequently, to develop professionals and specialists with the skills and political dispositions to recognize the relevance of the category’s representative entities and act, as a group, to promote improvements for nursing.

## CONCLUSION

The meanings female nurses attribute to work and politics in the era of liquid modernity reveal that the former is seen as a means of subsistence, i.e., it does not engage these workers’ subjectivities, and thus, there is a depletion of meanings related to labor. At the same time, they recognize that politics is important for achieving collective benefits, but they show no willingness to act in this perspective, as they consider it just another responsibility amidst the context of work intensification, and they view membership in professional bodies as an investment with little or no concrete return.

These findings reveal that female nurses are affected by individualism, immediacy, and superficiality as naturalized elements in contemporary society that reverberate in the workplace, weakening nursing and its organizations. Therefore, it is considered that these professionals have incorporated the precepts of neoliberal capitalism inaugurated by liquid modernity, because: they lack fulfillment in their work, since their activities are limited to receiving an wage and, therefore, do not produce subjectivities; they do not develop a sense of belonging to the class, which interferes with identity construction; and they distance themselves from political involvement, given the fluidity of relationships and the devaluation of collective struggles.

## DATA AVAILABILITY

The entire dataset supporting the results of this study was published in the article itself.

## References

[1] Fernandes FR, Gedrat DC, Vieira AG (2023). O significado do trabalho: um olhar
contemporâneo.. Cad Fucamp.

[2] Fontana CP (2021). A evolução do trabalho: da pré-história até ao
teletrabalho.. Rease.

[3] Dejours C, Zambroni-de-Souza PC, Barros VA (2023). Centralidade do trabalho e saúde mental.. Cad Psicol Soc Trab.

[4] Bauman Z (2021). Modernidade líquida.. São Paulo: Zahar.

[5] Mattiazzi A, Vila-Petroff M (2021). Is Bauman’s “liquid modernity” influencing the way we are doing
science?. J Gen Physiol..

[6] Silva TF, Aquino CAB, Lima IEP (2023). Liquid workers? Reflections on work from bauman’s
thought.. Rev.

[7] Arendt H (1998). O que é política?. Rio de Janeiro: Bertrand Brasil.

[8] Dias LB, Gondim DSG (2023). Política e pluralidade humana em Hannah Arendt: uma introdução ao
tema do poder.. Ágora Filos.

[9] Souza HS, Trapé CA, Campos CMS, Soares CB (2021). The Brazilian nursing workforce faced with the international
trends: an analysis in the International Year of Nursing.. Physis.

[10] Souza VR, Marziale MH, Silva GT, Nascimento PL (2021). Tradução e validação para a língua portuguesa e avaliação do guia
COREQ.. Acta Paul Enferm.

[11] Hennink M, Kaiser BN (2022). Sample sizes for saturati on in qualitative research: a
systematic review of empiricaltests.. Soc Sci Med.

[12] Carvalho DNR, Aguiar VFF, Apolinário DB, Bendelaque DFR, Pereira LCG, Figueira SAS (2024). A glanceat the use of IRaMuTeQ^®^ software in scientific
research: a bibliometric study.. Rev Enferm UFPI.

[13] Martins KN, Paula MC, Gomes LPS, Santos JE (2022). O software IRaMuTeQ como recurso para a análise textual
discursiva.. Rev Pesqui Qual.

[14] Bento LA, Lima MD, Borges MFC (2024). Análise de similitude utilizada para identificar relações entre
palavras dentro de um corpus textual.. Rease.

[15] Pinto LM, Andrade MHS, Campelo MV (2024). Análise com o software IRAMUTEQ^®^: estado do
conhecimento sobre percepção ambiental no ensino
fundamental.. RevENSIN.

[16] Carneiro RS, Lopes TB, Dias CMSL (2022). Ensino de matemática na revista prática docente: uma análise de
similitude com o uso do IRAMUTEQ.. Rev Prat Docente.

[17] Mattiazzi A, Vila-Petroff M (2021). Is Bauman’s “liquid modernity” influencing the way we are doing
science?. J Gen Physiol..

[18] Trevizan MB, Veras CA, Borges PP, Santos PHS (2023). ‘Ser leve e ser líquido’: a “modernidade líquida” no pensamento
de Zygmunt Bauman.. Synesis.

[19] Costa AA, Buckley D, Sousa APB, Figueira GL, Silva CNN (2024). O mundo do trabalho líquido e a realidade do trabalhador:
desafios da formação no contexto da precarização do trabalho a partir da
docência.. Multifaces.

[20] Lopes CB, Santos TL (2022). Diálogo entre Arendt e Bauman sobre os principais riscos à
democracia na modernidade líquida pós-pandemia: o cidadão transformado em
consumidor e a verdade factual ameaçada.. Estud Rev Multidiscip.

[21] Dias MO, Soares SSS, Gallasch CH, Carvalho EC, Melo NGS, Souza NVDO (2021). Lideranças de classe de enfermagem: seus papéis e tensionamentos
travados contra a precarização.. Belo Horizonte: Synapse.

[22] Farias SNP, Souza NVDO, Varella TCMML, Andrade KBS, Soares SSS, Carvalho EC (2023). Pejotização and implications for nursing work in Brazil:
repercussions of neoliberalism.. Rev Esc Enferm USP.

[23] Farias SNP, Souza NVDO, Andrade KBS, Varella TCML, Soares SSS, Carvalho EC (2021). Brazilian labor reform and implications for nursing work: a case
study.. Rev Esc Enferm USP.

[24] Han NK, Kim GS (2024). The barriers and facilitators influencing nurses’ political
participation or healthcare policy intervention: a systematic review and
qualitative meta-synthesis.. J Nurs Manag.

[25] Barzegar Safari M, Bahadori M, Alimohammadzadeh K (2020). The related factors of nurses’ participation and perceived
benefits and barriers in health policy making.. J Nurs Res.

