# Supplementary material for: Work and politics in the era of liquid modernity: meanings attributed by female nurses
Source: Rev Esc Enferm USP. 2025 Dec 12;59:e20250175. doi: 10.1590/1980-220X-REEUSP-2025-0175en (PMC12704942; doi:10.1590/1980-220X-REEUSP-2025-0175en)
Supplement: Translation

# Translation: Trabalho e política na era da modernidade líquida: sentidos atribuídos por enfermeiras^*^

**DOI:** 10.1590/1980-220X-REEUSP-2025-0175pt

**Authors:** Midian Oliveira Dias, Juliana Amaral Prata, Ana Beatriz Azevedo Queiroz, Carolina Cabral Pereira da Costa, Samira Silva Santos Soares, Luanny Regina de Oliveira Santos, Luana dos Santos Cunha de Lima, Norma Valéria Dantas de Souza

**Affiliations:** 1 Universidade do Estado do Rio de Janeiro, Faculdade de Enfermagem, Departamento de Enfermagem Materno-Infantil, Rio de Janeiro, RJ, Brasil.; 2 Universidade Federal do Rio de Janeiro, Escola de Enfermagem Anna Nery, Departamento de Enfermagem Materno-Infantil, Rio de Janeiro, RJ, Brasil.; 3 Universidade do Estado do Rio de Janeiro, Faculdade de Enfermagem, Departamento de Enfermagem Médico-Cirúrgico, Rio de Janeiro, RJ, Brasil.; 4 Universidade Estadual de Santa Cruz, Departamento de Ciências da Saúde, Ilhéus, BA, Brasil.; 5 Universidade Estácio de Sá, Rio de Janeiro, RJ, Brasil.; 6 Centro Federal de Educação Tecnológica Celso Suckow da Fonseca, Rio de Janeiro, RJ, Brasil.

**Keywords:** Enfermagem, Trabalho, Política, Engajamento no Trabalho, Capitalismo.

## Abstract

**Objetivo:**

compreender os sentidos atribuídos por enfermeiras ao trabalho e à política na modernidade líquida.

**Método:**

estudo qualitativo, com 46 estudantes de cursos da pós-graduação da faculdade de enfermagem de uma universidade pública estadual do município do Rio de Janeiro. Os dados foram coletados de abril a novembro de 2021, através de entrevistas individuais semiestruturadas, e submetidos ao *software* IRaMuTeQ^®^.

**Resultados:**

análise de similitude se estruturou em dois eixos centrais (“trabalho” e “política”), circundados por palavras periféricas. O primeiro emergiu como um meio de subsistência. O segundo é visto como mais uma atribuição em meio à intensificação do trabalho, que afasta as enfermeiras da atuação política, ainda que reconheçam sua importância no alcance de benefícios coletivos. Sobre as entidades de classe, vislumbram-nas como um investimento financeiro com baixo ou nenhum retorno concreto.

**Conclusão:**

referente à modernidade líquida, há um esvaziamento de significados associados ao trabalho concomitante à desvalorização das entidades de classe, culminando no afastamento da atuação política das enfermeiras.

## INTRODUÇÃO

O trabalho pode ser conceituado como qualquer atividade que necessita de gasto de energia física e/ou mental, de maneira direta ou indireta, com a finalidade de produzir bens e/ou serviços, sendo permeado de sentidos e valores de acordo com os contextos culturais e históricos que os permeiam. Além disso, tem a capacidade de contribuir na construção das subjetividades e identidades pessoais e coletivas, bem como oferece garantias de subsistência, utilidade, segurança, pertencimento a um grupo, *status* social, entre outros^(1,2,3)^.

No entanto, na contemporaneidade, houve uma mudança de paradigma no modo das pessoas serem e existirem no mundo, com a consequente transformação da relação do homem com o trabalho, sendo esse fenômeno descrito pelo sociólogo Zygmunt Bauman como modernidade líquida. Com a incorporação do modelo político-econômico-ideológico capitalista neoliberal, o trabalho passou a assumir uma vertente fluida, flexível e precária, perdendo a centralidade na vida do homem, e o consumo passou a ocupar este lugar^(4,5)^.

Em alusão metafórica ao estado líquido da matéria, que é facilmente moldado e adaptável às diferentes condições ambientais, esse rearranjo social complexo na atualidade se encontra instável e com dimensões indefinidas^(4,5,6)^. Ademais, há superficialidade e flexibilização das relações sociais, aceleração do ritmo de vida, impessoalidade, individualismo, consumismo e propensão às mudanças rápidas, conforme as nuances políticas e sociais^(4,5,6)^.

Esse contexto vai contra a natureza do ser humano, que é permeada por elementos essencialmente políticos e sociais, os quais vêm sendo desconstruídos pela modernidade líquida. No âmbito social, o consumo promove a instrumentalização das relações sociais, reduzindo-as a relações de consumo. Assim, há objetificação do outro, consumindo-se a utilidade e descartando-o quando não mais puder satisfazer os desejos individuais^(4,5,6)^.

Compreendendo a política como o meio pelo qual as pessoas buscam resolver conflitos e dispor a vida em sociedade, cabe ponderar que a modernidade líquida tem remodelado o existir humano em suas diversas facetas, a partir da valorização exacerbada do individualismo em detrimento do coletivo, com repercussões diretas sobre as formas de participação política, inclusive no campo do trabalho^(7,8)^.

Diante dessa compreensão, e transportando-a para o universo particular da enfermagem, há um consenso na literatura científica que descortina a fuga e o desinteresse da categoria em discutir política nos ambientes laborais. Por isso, acredita-se que a modernidade líquida alterou, significativamente, as relações da enfermagem e da sociedade de maneira geral com a política^(4,5,6)^.

Bauman classifica o individualismo, o imediatismo e o consumo como motivos para o afastamento do ser humano da política. Já que pessoas egocêntricas não estão dispostas a dedicar energia e tempo na busca por soluções para problemas coletivos, que exigem coragem e dedicação, assim como os resultados, em geral, são de longo prazo e não satisfazem os desejos individuais rapidamente^(4,5,6)^.

Tendo em vista que esse contexto conduz à perda de sentidos no trabalho decorrente do esvaziamento de significados associados ao labor e da diluição de sentimentos de classe, faz-se necessário entender as nuances dessas transformações, a fim de impulsionar reflexões junto ao coletivo profissional no que tange ao desenvolvimento de estratégias de enfrentamentos e superação^(3,4)^.

Nesse contexto, emerge a importância do presente estudo, sobretudo considerando que, no Brasil, a realidade laboral das enfermeiras pode corroborar para a apatia política, visto que essas trabalhadoras convivem com baixos salários, acúmulo de vínculos e jornadas extenuantes desenvolvidas em meio à polivalência, à intensificação e às inadequações nas condições de trabalho^(9)^. Diante desses apontamentos, o presente estudo objetivou compreender os sentidos atribuídos por enfermeiras ao trabalho e à política na era da modernidade líquida.

## MÉTODO

### Tipo de Estudo

Estudo de abordagem qualitativa que seguiu os critérios estabelecidos no *Consolidated criteria for Reporting Qualitative research* ^(10)^.

### Local, População e Critérios de Seleção

O cenário do estudo foi uma faculdade de enfermagem vinculada a uma universidade pública estadual localizada no município do Rio de Janeiro, Brasil. Os participantes da pesquisa foram 46 enfermeiros inscritos nos cursos de pós-graduação *lato sensu* e *stricto sensu* da referida instituição. O total de alunos inscritos, no período da coleta de dados, foi de 203, sendo 157 de pós-graduação *lato* e 46 de pós-graduação *stricto sensu*.

Os critérios de inclusão dos participantes foram estar regularmente matriculados nos cursos de pós-graduação durante o período de coleta de dados e ter, no mínimo, um ano de experiência profissional na enfermagem, garantindo, assim, a compreensão das especificidades do mundo do trabalho em enfermagem. Os critérios de exclusão foram ser profissionais que estavam inseridos no mercado de trabalho em áreas não relacionadas diretamente à enfermagem e residentes de enfermagem, pois esta modalidade de formação requer dedicação exclusiva, impossibilitando a inserção concomitante no mercado de trabalho.

### Coleta de Dados

A captação dos participantes se iniciou com um levantamento dos estudantes matriculados nos cursos de pós-graduação *lato* e *stricto sensu* junto às respectivas secretarias acadêmicas. Na sequência, a divulgação da pesquisa aconteceu por meio de um formulário digital, elaborado e hospedado na nuvem de um site gratuito, onde constavam os objetivos e o convite à participação no estudo, no qual os interessados informaram alguns dados para contato posterior e registraram a intenção de participar.

Como estratégia adicional, a autora responsável pela coleta de dados também utilizou a abordagem dos discentes em aulas remotas desenvolvidas por mediação tecnológica, haja vista as restrições à presencialidade do contexto pandêmico da COVID-19. Nessas ocasiões, os coordenadores dos cursos disponibilizaram alguns minutos para a apresentação dos objetivos do estudo e de sua relevância para a enfermagem, seguida do convite à participação na pesquisa.

Ao final, o processo de recrutamento resultou na captação de 46 enfermeiros, dos quais 34 foram recrutados através do formulário e 12 por abordagem oral. Destaca-se que não houve recusa à participação, mas houve sete perdas devido à baixa qualidade do áudio da gravação da entrevista.

Os dados foram coletados de abril a novembro de 2021 por meio de entrevistas individuais, que seguiram um roteiro semiestruturado, dividido em duas partes. A primeira parte continha dez perguntas fechadas para uma breve caracterização dos participantes (sexo, identidade de gênero, idade, curso, estado civil, número de filhos, responsabilidade pelo sustento da família, tipo e quantidade de vínculos empregatícios, associação do emprego com outras atividades remuneradas); e a segunda parte continha a seguinte questão disparadora: qual é a sua percepção, como enfermeira, sobre a relação entre política e trabalho?

As entrevistas foram realizadas por uma das autoras utilizando a ligação telefônica de voz, acontecendo na presença da referida pesquisadora e do participante. Tiveram duração média de 30 minutos, foram gravadas em áudio MP4, mediante autorização prévia, e transcritas na sequência. Cabe esclarecer que as entrevistas foram conduzidas por uma das autoras, enfermeira, docente, doutoranda em enfermagem na época da coleta de dados e com experiência na produção de dados qualitativos. Ainda, a mesma realizou a testagem do instrumento com duas doutorandas, as quais não foram incluídas no estudo.

A coleta de dados foi encerrada quando o material transcrito não apresentou ideias novas, entendendo, assim, que se alcançou a saturação temática indutiva^(11)^, a qual foi identificada na quadragésima terceira entrevista, sendo confirmada com a realização de mais três. Ademais, todas as participantes receberam a transcrição da sua entrevista para confirmação das informações, mas não houve qualquer retorno por parte delas com solicitações para alterações.

### Análise e Tratamento Dos Dados

Para análise de dados, utilizou-se o *software Interface de R pourles Analyses Multidimensionsionnelles de Textes et de Questionnaires* (IRaMuTeQ^®^), que é um programa livre, gratuito e ancorado no *software* R (pacote gratuito para realizar análises estatísticas), possibilitando diferentes processamentos e análises estatísticas dos textos.

Para fins de esclarecimento, os textos originados do conjunto de entrevistas são denominados *corpus* de análise. Cada entrevista isolada é chamada de “texto”, e os excertos de texto (enunciados, ambiente das palavras) são nominados de “segmentos de texto” (STs)^(12,13)^. Dessa forma, as 46 entrevistas foram transcritas e agrupadas em um único documento de texto eletrônico, com 225 páginas, sendo que o *corpus* da pesquisa teve 46 textos e 2.554 STs, dos quais 2.362 foram aproveitados, representando um aproveitamento satisfatório do material com retenção de 92,48%.

Para o processamento de dados, optou-se pela análise de similitude, que agrupa as palavras, organizando-as graficamente em função da sua frequência. Essa técnica é baseada na teoria dos grafos, e possibilita observar as ocorrências e coocorrências entre os léxicos, bem como indica conectividades entre os mesmos, contribuindo para o reconhecimento da estrutura dos conteúdos e núcleos temáticos que auxiliam na resposta ao objetivo do estudo^(12,13,14)^.

Além disso, utilizaram-se o referencial teórico proposto por Bauman^(4)^ sobre modernidade líquida e produções científicas sobre o tema trabalho em enfermagem.

### Aspectos Éticos

A pesquisa foi aprovada pelo Comitê de Ética em Pesquisa da Universidade do Estado do Rio de Janeiro, sob Parecer n° 4.145.807 e Certificado de Apresentação de Apreciação Ética n° 32251320.0.0000.5282 em 09 de julho de 2020. O estudo atendeu aos aspectos éticos de pesquisas com seres humanos. Os participantes assinaram o Termo de Consentimento Livre e Esclarecido, tendo a sua identidade preservada por meio de uma codificação, tal como o uso da letra “E”, que representa enfermeiro, seguida de um algarismo correspondente à ordem de realização das entrevistas.

## RESULTADOS

Participaram deste estudo 46 enfermeiros, alunos de cursos de pós-graduação, sendo que 34 cursavam a pós-graduação na modalidade *lato sensu*, seis, o curso de mestrado, e seis, de doutorado. A maioria se identifica como mulher (40), seis, como homem, e todas as pessoas participantes se declararam cisgênero, com idade variando entre 30 e 35 anos. A partir desse achado, optou-se por tratar o conjunto de participantes no feminino, compreendendo que a enfermagem é uma profissão majoritariamente feminina e o maior quantitativo de participantes é de mulheres.

No tocante ao estado civil, 17 são solteiras; 18 são casadas; três declararam ser divorciadas; quatro se encontravam em união estável; e quatro não responderam. Além disso, 25 não tinham filhos e 21 possuíam filhos. Quanto à renda, 13 participantes não eram responsáveis pelo sustento da família, enquanto que 24 possuíam essa responsabilidade e nove responderam que talvez, ou algumas vezes exerciam a incumbência de prover financeiramente a casa/família.

Em relação ao vínculo empregatício, 29 relatam ter um emprego formal, entre as quais: sete associavam essa vinculação com uma atividade remunerada informal; oito possuíam dois vínculos formais, sendo que duas associavam o duplo vínculo com uma atividade remunerada informal; quatro possuíam três ou mais vínculos formais, das quais duas somavam esta condição com uma atividade remunerada informal; três não responderam; e duas estavam recém desligadas do vínculo formal.

Na análise de similitude, o *software* gerou a Figura 1, que apresenta dois eixos centrais (“política” e “trabalho”), circundados por palavras periféricas.

**Figura 1:** Análise de similitude e relações entre as palavras “política” e “trabalho”

Fonte: dados do IRaMuTeQ^®^. Rio de Janeiro, Brasil, 2025.

A fim de elucidar as representações contidas na árvore de similitude, alguns STs descrevem a compreensão das participantes acerca da política no contexto laboral como essencial para o alcance de benefícios. No entanto, nota-se que elas se afastam de assuntos relacionados à mesma, pois a consideram como mais uma atribuição no ambiente do trabalho.

*A política está presente no nosso dia a dia de trabalho na saúde. O enfermeiro sempre acaba fugindo dessa responsabilidade, como se a participação política fosse mais um peso que ele teria que carregar. Mais um dever!* (E25)

*Acredito que, para conseguir benefício no trabalho, é com a política. Ela está presente em todas as relações da enfermagem, mas, mesmo sabendo disso, não nos envolvemos com a política, pois já estamos sobrecarregadas de deveres.* (E12)

*Para melhoria do meu trabalho, é com a política. Quando acontecem problemas, me torno mais ativa politicamente, mas, no dia a dia, como enfermeira, eu não me envolvo em questões políticas, pois já tenho muitas atribuições.* (E08)

Além disso, como um sentido atribuído ao trabalho, os STs descortinam que as enfermeiras vêem o labor como um meio de garantir a subsistência, a partir da obtenção de um salário.

*Nós trabalhamos e lutamos pela sobrevivência. Pagar as contas e ter dinheiro é a prioridade. É a finalidade do trabalho! Nossa carga horária já é excessiva! Não queremos nos envolver politicamente.* (E41)

*Eu amo a enfermagem. Amo prestar assistência ao paciente. Mesmo assim, eu trabalho por dinheiro, pela remuneração… trabalhamos com amor e por dinheiro.* (E03)

*Tenho amor pela minha profissão, mas amor não paga as contas. Precisamos do salário! O trabalho é difícil, as relações com paciente, com os colegas, com a chefia são difíceis. Permaneço no hospital porque preciso do salário, da remuneração para sobreviver.* (E30)

Outrossim, alguns STs demarcam a relação de distanciamento das trabalhadoras com suas entidades representativas da categoria de enfermagem, uma vez que consideram os pagamentos a essas organizações como um ônus sem o retorno desejado.

*A classe está abandonada, trabalhando fora das condições ideais porque precisamos de dinheiro para sobreviver. Não temos representatividade. Para se sindicalizar, é necessário pagar! Os valores cobrados acabam pesando no orçamento e, ainda, têm baixo retorno e distanciamento da categoria. Isso desestimula.* (E12)

*Não vejo acontecer! Não vi o retorno que eu queria por parte do sindicato. Paguei por anos e, até hoje, não vi nenhum benefício. Nada mudou. As entidades representativas de classe estão muito distantes da categoria. Elas não sabem dos nossos problemas e do que precisamos.* (E08)

*Vejo muitos colegas dizendo que o COREN e o sindicato não estão fazendo nada. Eles só querem pagamentos todo ano e não fazem nada. Esse é o discurso da nossa classe.* (E18)

## DISCUSSÃO

Na análise de similitude, o tamanho dos vocábulos e a espessura dos traços que os unem representam o quanto os termos são importantes para compreensão do fenômeno estudado^(15,16)^. Neste sentido, sob a vertente de dois eixos semânticos, evidenciouse que, no primeiro, “política” é uma palavra central circundada perifericamente por “participação política”, “dever”, “direito”, “envolver”, “sociedade”, “saúde” e outros vocábulos. Já no segundo, o léxico “trabalho” apareceu como central e se relaciona com os seguintes termos periféricos: “carga horária”, “remuneração”, “acontecer”, “coren”, “sindicato”, “classe”, dentre outros.

Nessa perspectiva analítica, os léxicos centrais “política” e “trabalho” são as palavras que possuem as linhas de ligação com maiores espessuras entre si, indicando maior coocorrência (aparecimento dessas palavras de forma conjunta), como quando as enfermeiras relatam que a política está presente no cotidiano laboral da enfermagem. Ao mesmo tempo, há um distanciamento entre essas palavras centrais, revelando que a política ainda não é um elemento que compõe as ações intencionais diárias da enfermagem, haja vista que os STs mostram atitudes de fuga e distanciamento diante de temas relacionados à política, vislumbrando-a como mais uma atribuição em um cenário de sobrecargas e longas jornadas no ambiente do trabalho.

Outrossim, os STs conduzem à compreensão de que o labor é apenas um meio de subsistência na modernidade líquida e, considerando os significados de trabalho descritos por Bauman, duas realidades opostas são confrontadas. A primeira, denominada de modernidade sólida, caracterizava-se por um ordenamento social advindo da aliança entre os Estados-nação e a ciência. Nesse cenário, os trabalhadores possuíam baixa mobilidade e escolaridade, mas as relações com o trabalho eram duradouras e formais, sendo comum ingressar no mercado de trabalho e se aposentar no mesmo vínculo ou função. Além disso, o trabalho proporcionava subsistência financeira, agregando *status* social, seguridade, satisfação, relações interpessoais e outros elementos essenciais ao ser humano e à constituição da identidade^(4,5,6, 17, 18)^.

Já a segunda realidade, identificada entre as participantes deste estudo, surgiu a partir de mudanças decorrentes de crises que transformaram a conjuntura social, política e econômica, como as tragédias nucleares, os campos de concentração e o Holocausto, ruindo as estruturas da modernidade sólida. Como efeitos, inaugurou-se a modernidade líquida enquanto uma lógica que demarca as diferenças e difunde ideais de individualização, liberdade, eliminação da sociabilidade e dependência, opondo-se, assim, ao paradigma da modernidade sólida que a antecedeu^(4,5,6, 18)^.

Essa nova modernidade adveio das ideias de fluidez, leveza, precariedade, incerteza e inconstância, trazendo consigo as noções de praticidade, adaptabilidade e superficialidade das relações, além da aceleração do ritmo de vida e exacerbação da liberdade, do individualismo, do consumismo e da flexibilidade. Concomitantemente, verificam-se a expansão do capitalismo neoliberal e o enfraquecimento da relação entre capital, trabalho e Estado, com o intuito de favorecer a reprodução do capital de forma autônoma, globalizada e livre. Por conseguinte, o Estado se tornou mínimo e o trabalho flexível e precário^(4,5,6, 18)^.

No mundo do trabalho, essas questões se traduzem na busca por trabalhadores especializados e multifuncionais com relações laborais precárias e instáveis, de modo que permanecer por muito tempo no mesmo emprego ou função é difícil e, quando ocorre, não é bem visto, pois reflete a estagnação e não progressão do mercado. Diante disso, o emprego informal se expande, e a identidade profissional se torna passageira, reconstruindo-se constantemente a partir de estímulos relacionados ao consumo^(4,5,6, 19)^.

Considerando que a pessoa é o que ela consome ou possui, e que o consumo passou a ocupar o lugar antes preenchido pelo trabalho e pelas relações interpessoais, tem-se que, na modernidade líquida, a identidade e as subjetividades do indivíduo assumem caráter fluído^(16,17,18,19)^. Nesse sentido, compreende-se o porquê de os sentidos do trabalho para as enfermeiras entrevistadas serem voltados para ganhos monetários, que garantem a subsistência e o consumo, de modo que se envolver com questões políticas em prol de ganhos coletivos não é uma prioridade. Consequentemente, a perda de sentido do trabalho está alinhada ao esvaziamento de significados, devido às elevadas demandas modernas líquidas e ao consumo^(19)^.

No âmbito da enfermagem, esse contexto laboral sombrio se agrava pelas características sociodemográficas da categoria, além de questões de gênero, origem histórica e herança cultural da profissão, que convivem com sobrecargas de tarefas no turno de trabalho, extensas jornadas, baixos salários, e desvalorização social e profissional^(4,5,6)^. Na contemporaneidade, essa conjuntura conduz as enfermeiras à apatia política, conforme evidenciado nesta pesquisa, por meio das subjetividades relacionadas ao trabalho, haja vista a priorização do consumo, que pode desencadear a fluidez da identidade dessas trabalhadoras.

Corroborando, destaca-se a opção das participantes pelo afastamento dos espaços de discussão e luta política, apesar de reconhecerem a importância desse envolvimento para a conquista de melhorias nas condições laborais. Na verdade, esse resultado revela o individualismo, onde as aspirações pessoais estão acima das coletivas, e o imediatismo, que enfraquece a consciência de classe como elementos naturalizados pela modernidade líquida e presentes em todas as esferas da vida, incluindo o trabalho^(4,5,6, 16,17,18,19)^.

Considerando a extremidade do segundo núcleo de sentido apresentado na árvore de similitude, nota-se que o léxico “coren” está próximo de “classe” e “sindicato”, indicando a correlação entre essas entidades representativas da profissão e da categoria. Essas palavras são pertencentes ao leque semântico do “trabalho”, porém estão perifericamente distantes, apresentando uma linha de ligação frágil com o termo central. Assim, o distanciamento entre esses léxicos demarca que não há coocorrência significativa.

De igual modo, os STs revelam que, na visão das enfermeiras, as entidades representativas da enfermagem não se apropriam das reais necessidades da categoria por não possuírem relações estreitas com as trabalhadoras que a compõem. Assim, vislumbram os valores cobrados por essas organizações como uma barreira de aproximação e que esses gastos impactam o orçamento. Em contrapartida, há percepção de que a filiação, a inscrição e a associação se revertem em baixo ou nenhum retorno em termos de benefícios coletivos aos profissionais advindos das lutas políticas. Dessa forma, conforma-se um ciclo de afastamento e sentimento de ausência de representatividade, que é acompanhado pelo enfraquecimento da entidade de classe e pelo consequente aumento do distanciamento.

Em outra perspectiva, pondera-se que as entidades representativas da enfermagem representam o espaço público, o qual não é valorizado na modernidade líquida, haja vista que as pessoas são conduzidas a se manter ocupadas e preocupadas, cada vez mais, com suas próprias esferas privadas. Nesse sentido, por meio da disseminação do individualismo, o eu cidadão é substituído pelo eu indivíduo, de modo que, quando o espaço público é ocupado apenas pelo eu indivíduo, o objetivo coletivo acaba sendo desviado para um propósito individual. Assim, os pensamentos e as ações coletivas deixam de existir, e as iniciativas de publicização da vida privada acontecem, levando o grupo à alienação(4,5,6, 19, 20).

Nesse contexto, somam-se a superficialidade das relações com os pares, que impede a construção de interações profundas e empáticas, e a centralidade do consumo, enquanto um componente essencial da identidade da pessoa que, ao reduzir a noção de cidadania, coloca o “ser consumidor” acima do “ser humano”. Ademais, a carência de relações sociais genuínas e a troca entre a vida pública e privada, associadas ao egocentrismo e à insensibilidade, impulsionadas pela modernidade líquida^(4,5,6, 19, 20)^, formam um panorama que distancia os enfermeiros da esfera política e, consequentemente, da participação nesse espaço e da possibilidade de constituir uma relação próxima e verdadeira com as entidades representativas da profissão.

Outrossim, ressalta-se a importância de os profissionais compreenderem as competências legais distintas de cada uma dessas organizações, para que busquem informações e auxílio junto aos canais de comunicação apropriados à natureza de suas demandas^(21)^. Para tanto, é preciso que as trabalhadoras da enfermagem rompam com a alienação, imposta pelo trabalho na era da modernidade líquida, e se aproximem das suas entidades representativas por meio da filiação, participação em assembleias e eventos científicos, culturais, sociais e políticos, além de elaboração de pareceres e compartilhamento de opiniões individuais sobre assuntos publicados na mídia. Tais estratégias possibilitam o exercício do controle social, aproximam os profissionais de seus pares e fortalecem as instituições próprias da profissão.

Essas questões são fundamentais, pois, na atualidade, muitas lutas no âmbito do trabalho culminam em disputas judiciais, que se deparam com a burocratização e a morosidade do sistema judiciário. Além disso, esses processos se ancoram em legislações que são tendenciosas ao favorecimento do capital em detrimento ao trabalhador. É o caso, por exemplo, das reformas trabalhistas e previdenciárias, ocorridas nos anos de 2017 e 2019, que, apesar de trazerem o discurso da ampliação do emprego e da flexibilização das jornadas de trabalho, na realidade, conformaram-se como movimentos de retirada de direitos da classe trabalhadora^(21,22,23)^.

Nesse contexto, é premente que os profissionais de enfermagem e as entidades representativas da profissão se organizem enquanto um coletivo para que as suas lutas conquistem espaços de poder e decisão potentes para impulsionar mudanças concretas na realidade laboral das enfermeiras em meio às relações de força da política, da economia e do trabalho na modernidade líquida.

Entende-se que a limitação do estudo reside em sua abrangência, visto que foi realizado em apenas uma instituição de ensino superior do estado do Rio de Janeiro, o qual tem especificidades culturais, políticas e do próprio campo da saúde que não necessariamente se apresentam em outros estados brasileiros. Portanto, os resultados podem não ser generalizáveis, mas se alinham com os achados de pesquisas internacionais que mostram o baixo envolvimento da enfermagem com temas relacionados à política^(24,25)^.

Ademais, este estudo oferece subsídios para reflexões críticas acerca das repercussões da modernidade líquida sobre a configuração laboral, a enfermagem enquanto um coletivo profissional e as atitudes individuais em relação às lutas coletivas no campo político. Destaca-se que essa problematização deve acontecer nos espaços coletivos do trabalho, porém não se limita aos trabalhadores. É urgente que esse debate permeie os processos de ensino e aprendizagem da graduação até a pós-graduação, no sentido de oportunizar uma formação contextualizada com o trabalho e, consequentemente, a constituição de profissionais e especialistas com competências e disposições políticas para reconhecer a relevância das entidades representativas da categoria e agir, enquanto grupo, em prol de melhorias para a profissão.

## CONCLUSÃO

Os sentidos atribuídos por enfermeiras ao trabalho e à política na era da modernidade líquida revelam que o primeiro é visto como um meio de subsistência, ou seja, não mobiliza as subjetividades dessas trabalhadoras e, por isso, há um esvaziamento de significados relacionados ao labor. Ao mesmo tempo, reconhecem que a política é importante para o alcance de benefícios coletivos, mas não mostram disposições para agir nessa perspectiva, pois a consideram como mais uma atribuição em meio ao contexto de intensificação do trabalho, assim como vislumbram a filiação às entidades representativas da profissão como um investimento com baixo ou nenhum retorno concreto.

Esses achados desvelam que as enfermeiras são afetadas pelo individualismo, pelo imediatismo e pela superficialidade como elementos naturalizados na sociedade contemporânea que reverberam nos ambientes de trabalho, enfraquecendo a categoria e suas organizações. Diante disso, pondera-se que essas profissionais incorporaram os preceitos do capitalismo neoliberal inaugurado pela modernidade líquida, pois: não se realizam no labor, uma vez que suas atividades se limitam ao recebimento de um salário e, por isso, não produzem subjetividades; não desenvolvem o sentimento de pertencimento à classe, o que interfere na construção da identidade; e se distanciam do envolvimento político, haja vista a fluidez das relações e a desvalorização das lutas coletivas.

## DISPONIBILIDADE DE DADOS

Todo o conjunto de dados que dá suporte aos resultados deste estudo foi publicado no próprio artigo.

# Translation: Trabajo y política en la era de la modernidad líquida: significados atribuidos por las enfermeras^*^

**DOI:** 10.1590/1980-220X-REEUSP-2025-0175es

**Authors:** Midian Oliveira Dias, Juliana Amaral Prata, Ana Beatriz Azevedo Queiroz, Carolina Cabral Pereira da Costa, Samira Silva Santos Soares, Luanny Regina de Oliveira Santos, Luana dos Santos Cunha de Lima, Norma Valéria Dantas de Souza

**Affiliations:** 1 Universidade do Estado do Rio de Janeiro, Faculdade de Enfermagem, Departamento de Enfermagem Materno-Infantil, Rio de Janeiro, RJ, Brasil.; 2 Universidade Federal do Rio de Janeiro, Escola de Enfermagem Anna Nery, Departamento de Enfermagem Materno-Infantil, Rio de Janeiro, RJ, Brasil.; 3 Universidade do Estado do Rio de Janeiro, Faculdade de Enfermagem, Departamento de Enfermagem Médico-Cirúrgico, Rio de Janeiro, RJ, Brasil.; 4 Universidade Estadual de Santa Cruz, Departamento de Ciências da Saúde, Ilhéus, BA, Brasil.; 5 Universidade Estácio de Sá, Rio de Janeiro, RJ, Brasil.; 6 Centro Federal de Educação Tecnológica Celso Suckow da Fonseca, Rio de Janeiro, RJ, Brasil.

**Keywords:** Enfermería, Trabajo, Política, Compromiso Laboral, Capitalismo.

## Abstract

**Objetivo:**

comprender los significados que las enfermeras atribuyen al trabajo y a la política en la modernidad líquida.

**Métodos:**

estudio cualitativo con 46 estudiantes de posgrado de la facultad de enfermería de una universidad pública estatal de Río de Janeiro. Los datos se recopilaron de abril a noviembre de 2021 mediante entrevistas individuales semiestructuradas y se procesaron mediante el software IRaMuTeQ^®^.

**Resultados:**

el análisis de similitud se estructuró en torno a dos ejes centrales (“trabajo” y “política”), rodeados de términos periféricos. El primero surgió como medio de subsistencia. El segundo se percibe como un deber más en medio de la intensificación del trabajo, lo que distancia a las enfermeras de la actividad política, aun cuando reconocen su importancia para el logro de beneficios colectivos. En cuanto a las asociaciones profesionales, las perciben como una inversión financiera con escasa o nula rentabilidad.

**Conclusión:**

en cuanto a la modernidad líquida, se observa un agotamiento de los significados asociados al trabajo, concomitante con la devaluación de las asociaciones profesionales, que culmina en el retiro de las enfermeras de la actividad política.

## INTRODUCCIÓN

El trabajo puede conceptualizarse como cualquier actividad que requiera un gasto de energía física y/o mental, de manera directa o indirecta, con el fin de producir bienes y/o servicios, y que está impregnado de significados y valores de acuerdo con los contextos culturales e históricos que lo rodean.

Además, tiene la capacidad de contribuir a la construcción de subjetividades e identidades personales y colectivas, así como ofrece garantías de subsistencia, utilidad, seguridad, pertenencia a un grupo, *estatus* social, entre otros^(1,2,3)^.

Sin embargo, en la contemporaneidad, se ha producido un cambio de paradigma en la forma de ser y existir de las personas en el mundo, con la consiguiente transformación de la relación del hombre con el trabajo, fenómeno descrito por el sociólogo Zygmunt Bauman como modernidad líquida. Con la incorporación del modelo político-económico-ideológico capitalista neoliberal, el trabajo pasó a asumir una vertiente fluida, flexible y precaria, perdiendo su centralidad en la vida del hombre, y el consumo pasó a ocupar este lugar^(4,5)^.

En alusión metafórica al estado líquido de la materia, que se moldea fácilmente y se adapta a las diferentes condiciones ambientales, esta compleja reorganización social se encuentra actualmente inestable y con dimensiones indefinidas^(4,5,6)^. Además, hay superficialidad y flexibilización de las relaciones sociales, aceleración del ritmo de vida, impersonalidad, individualismo, consumismo y propensión a cambios rápidos, según los matices políticos y sociales^(4,5,6)^.

Este contexto va en contra de la naturaleza del ser humano, que está impregnada de elementos esencialmente políticos y sociales, los cuales han sido deconstruidos por la modernidad líquida. En el ámbito social, el consumo promueve la instrumentalización de las relaciones sociales, reduciéndolas a relaciones de consumo. Así, se produce una objetivación del otro, consumiendo su utilidad y descartándolo cuando ya no puede satisfacer los deseos individuales^(4,5,6)^.

Entendiendo la política como el medio por el cual las personas buscan resolver conflictos y disponer de la vida en sociedad, cabe considerar que la modernidad líquida ha remodelado la existencia humana en sus diversas facetas, a partir de la valoración exacerbada del individualismo en detrimento del colectivo, con repercusiones directas en las formas de participación política, incluso en el ámbito laboral^(7,8)^.

Ante esta comprensión, y trasladándola al universo particular de la enfermería, existe un consenso en la literatura científica que revela la huida y el desinterés de la categoría por discutir política en los entornos laborales. Por ello, se cree que la modernidad líquida ha alterado significativamente las relaciones de la enfermería y de la sociedad en general con la política^(4,5,6)^.

Bauman clasifica el individualismo, el inmediatismo y el consumo como motivos para el alejamiento del ser humano de la política. Dado que las personas egocéntricas no están dispuestas a dedicar energía y tiempo a la búsqueda de soluciones a problemas colectivos, que requieren valor y dedicación, y que los resultados, en general, son a largo plazo y no satisfacen rápidamente los deseos individuales^(4,5,6)^.

Teniendo en cuenta que este contexto conduce a la pérdida de sentido en el trabajo, debido al vaciamiento de significados asociados al trabajo y a la dilución de los sentimientos de clase, es necesario comprender los matices de estas transformaciones, con el fin de impulsar reflexiones junto al colectivo profesional en lo que se refiere al desarrollo de estrategias de enfrentamiento y superación^(3,4)^.

En este contexto, surge la importancia del presente estudio, sobre todo teniendo en cuenta que, en Brasil, la realidad laboral de las enfermeras puede corroborar la apatía política, ya que estas trabajadoras conviven con salarios bajos, acumulación de vínculos y jornadas agotadoras desarrolladas en medio de la polivalencia, la intensificación y las inadecuaciones en las condiciones de trabajo^(9)^. Ante estas observaciones, el presente estudio tuvo como objetivo comprender los significados que las enfermeras atribuyen al trabajo y a la política en la era de la modernidad líquida.

## MÉTODO

### Tipo de Estudio

Estudio de enfoque cualitativo que siguió los criterios establecidos en los Criterios consolidados para la presentación de informes de investigación cualitativa (*Consolidated criteria for Reporting Qualitative research)* ^(10)^.

### Lugar, Población y Criterios de Selección

El escenario del estudio fue una facultad de enfermería vinculada a una universidad pública estatal ubicada en el municipio de Río de Janeiro, Brasil. Los participantes en la investigación fueron 46 enfermeros matriculados en los cursos de posgrado *lato sensu* y *stricto sensu* de dicha institución. El total de estudiantes matriculados, en el período de recolección de datos, fue de 203, siendo 157 de posgrado *lato* y 46 de posgrado *stricto sensu*.

Los criterios de inclusión de los participantes fueron estar matriculados regularmente en los cursos de posgrado durante el periodo de recolección de datos y tener, como mínimo, un año de experiencia profesional en enfermería, garantizando así la comprensión de las especificidades del mundo laboral en enfermería. Los criterios de exclusión fueron ser profesionales que estuvieran insertados en el mercado laboral en áreas no relacionadas directamente con la enfermería y residentes de enfermería, ya que esta modalidad de formación requiere dedicación exclusiva, lo que imposibilita la inserción concomitante en el mercado laboral.

### Recolección de Datos

La captación de participantes comenzó con un estudio de los estudiantes matriculados en cursos de posgrado *lato* y *stricto sensu* en las respectivas secretarías académicas. A continuación, la divulgación de la investigación se realizó mediante un formulario digital, elaborado y alojado en la nube de un sitio web gratuito, en el que se incluían los objetivos y la invitación a participar en el estudio, en el que los interesados proporcionaban algunos datos para su posterior contacto y registraban su intención de participar.

Como estrategia adicional, la autora responsable de la recolección de datos también utilizó el enfoque de los estudiantes en clases remotas desarrolladas por mediación tecnológica, dadas las restricciones a la presencialidad del contexto pandémico de la COVID-19. En esas ocasiones, los coordinadores de los cursos dedicaron unos minutos a presentar los objetivos del estudio y su relevancia para la enfermería, seguido de la invitación a participar en la investigación.

Al final, el proceso de reclutamiento dio como resultado la captación de 46 enfermeros, de los cuales 34 fueron reclutados a través del formulario y 12 por vía oral. Cabe destacar que no hubo rechazos a la participación, pero hubo siete pérdidas debido a la baja calidad del audio de la grabación de la entrevista.

Los datos se recopilaron de abril a noviembre de 2021 mediante entrevistas individuales, que siguieron un guion semiestructurado, dividido en dos partes. La primera parte contenía diez preguntas cerradas para una breve caracterización de los participantes (sexo, identidad de género, edad, carrera, estado civil, número de hijos, responsabilidad por el sustento de la familia, tipo y cantidad de vínculos laborales, asociación del empleo con otras actividades remuneradas); y la segunda parte contenía la siguiente pregunta desencadenante: ¿cuál es su percepción, como enfermera, sobre la relación entre política y trabajo?

Las entrevistas fueron realizadas por una de las autoras mediante llamada telefónica, en presencia de dicha investigadora y del participante. Tuvieron una duración media de 30 minutos, se grabaron en audio MP4, previa autorización, y se transcribieron a continuación. Cabe aclarar que las entrevistas fueron realizadas por una de las autoras, enfermera, profesora y doctoranda en enfermería en el momento de la recopilación de datos, con experiencia en la producción de datos cualitativos. Además, ella misma realizó la prueba del instrumento con dos doctorandas, que no fueron incluidas en el estudio.

La recolección de datos finalizó cuando el material transcrito no presentó ideas nuevas, entendiendo así que se había alcanzado la saturación temática inductiva^(11)^, que se identificó en la entrevista número cuarenta y tres y se confirmó con la realización de tres más. Además, todas las participantes recibieron la transcripción de su entrevista para confirmar la información, pero no hubo ninguna respuesta por su parte con solicitudes de cambios.

### Análisis y Tratamiento de Los Datos

Para el análisis de los datos se utilizó el software Interface de R pour les Analyses Multidimensionnelles de Textes et de Questionnaires (IRaMuTeQ^®^), que es un programa libre, gratuito y basado en el software R (paquete gratuito para realizar análisis estadísticos), que permite diferentes procesamientos y análisis estadísticos de los textos.

A efectos aclaratorios, los textos procedentes del conjunto de entrevistas se denominan *corpus* de análisis. Cada entrevista aislada se denomina “texto”, y los extractos de texto (enunciados, entorno de las palabras) se denominan “segmentos de texto” (ST)^(12,13)^. De esta forma, las 46 entrevistas fueron transcritas y agrupadas en un único documento de texto electrónico, con 225 páginas, y el corpus de la investigación contó con 46 textos y 2554 ST, de los cuales se utilizaron 2362, lo que representa un aprovechamiento satisfactorio del material, con una retención del 92,48%.

Para el procesamiento de los datos, se optó por el análisis de similitud, que agrupa las palabras y las organiza gráficamente en función de su frecuencia. Esta técnica se basa en la teoría de grafos y permite observar las ocurrencias y coocurrencias entre los léxicos, así como indicar las conectividades entre ellos, lo que contribuye al reconocimiento de la estructura de los contenidos y los núcleos temáticos que ayudan a responder al objetivo del estudio^(12,13,14)^.

Además, se utilizó el marco teórico propuesto por Bauman^(4)^ sobre la modernidad líquida y las producciones científicas sobre el tema del trabajo en enfermería.

### Aspectos Éticos

La investigación fue aprobada por el Comité de Ética en Investigación de la Universidad Estatal de Río de Janeiro, bajo el dictamen n.º 4.145.807 y el Certificado de Presentación de Evaluación Ética n.º 32251320.0.0000.5282, el 9 de julio de 2020. El estudio cumplió con los aspectos éticos de las investigaciones con seres humanos. Los participantes firmaron el Término de Consentimiento Libre e Ilustrado, y su identidad se preservó mediante una codificación, como el uso de la letra “E”, que representa enfermero, seguida de un número correspondiente al orden de realización de las entrevistas.

## RESULTADOS

Participaron en este estudio 46 enfermeros, estudiantes de cursos de posgrado, de los cuales 34 cursaban un posgrado en la modalidad *lato sensu*, seis, un curso de maestría, y seis, un doctorado. La mayoría se identificó como mujer (40), seis como hombre, y todos los participantes se declararon cisgénero, con edades comprendidas entre los 30 y los 35 años. A partir de este hallazgo, se optó por tratar al conjunto de participantes en femenino, entendiendo que la enfermería es una profesión mayoritariamente femenina y que la mayor parte de los participantes son mujeres.

En cuanto al estado civil, 17 son solteras; 18 están casadas; tres declararon estar divorciadas; cuatro se encontraban en unión libre; y cuatro no respondieron. Además, 25 no tenían hijos y 21 tenían hijos. En cuanto a los ingresos, 13 participantes no eran responsables del sustento de la familia, mientras que 24 tenían esa responsabilidad y nueve respondieron que tal vez, o en algunas ocasiones, ejercían la tarea de proveer económicamente al hogar/familia.

En relación con el vínculo laboral, 29 informaron tener un empleo formal, entre las cuales: siete asociaban ese vínculo con una actividad remunerada informal; ocho tenían dos vínculos formales, de las cuales dos asociaban el doble vínculo con una actividad remunerada informal; cuatro tenían tres o más vínculos formales, de las cuales dos sumaban esta condición con una actividad remunerada informal; tres no respondieron; y dos acababan de dejar el vínculo formal.

En el análisis de similitud, el software generó la Figura 1, que presenta dos ejes centrales (“política” y “trabajo”), rodeados por palabras periféricas.

**Figura 1:** Análisis de similitud y relaciones entre las palabras “política” y “trabajo”

Fuente: datos del IRaMuTeQ^®^. Río de Janeiro, Brasil, 2025.

Con el fin de aclarar las representaciones contenidas en el árbol de similitud, algunos ST describen la comprensión de las participantes sobre la política en el contexto laboral como esencial para obtener beneficios. Sin embargo, se observa que se alejan de los temas relacionados con ella, ya que la consideran una tarea más en el entorno laboral.

*La política está presente en nuestro día a día en el trabajo en el ámbito de la salud. El enfermero siempre acaba eludiendo esta responsabilidad, como si la participación política fuera una carga más que tendría que soportar. ¡Una obligación más!* (E25)

*Creo que, para obtener beneficios en el trabajo, hay que recurrir a la política. Está presente en todas las relaciones de la enfermería, pero, aun sabiéndolo, no nos involucramos en la política, ya que estamos sobrecargadas de obligaciones*. (E12)

*Para mejorar mi trabajo, hay que recurrir a la política. Cuando surgen problemas, me vuelvo más activa políticamente, pero en el día a día, como enfermera, no me involucro en cuestiones políticas, ya que tengo muchas responsabilidades.* (E08)

Además, como sentido atribuido al trabajo, los ST revelan que las enfermeras ven el trabajo como un medio para garantizar su subsistencia, a partir de la obtención de un salario.

*Trabajamos y luchamos por sobrevivir. Pagar las cuentas y tener dinero es la prioridad. ¡Es el objetivo del trabajo! ¡Nuestra carga horaria ya es excesiva! No queremos involucrarnos políticamente.* (E41)

*Amo la enfermería. Amo atender a los pacientes. Aun así, trabajo por dinero, por la remuneración... trabajamos con amor y por dinero*. (E03)

*Amo mi profesión, pero el amor no paga las cuentas. ¡Necesitamos el salario! El trabajo es difícil, las relaciones con los pacientes, con los compañeros, con los jefes son difíciles. Me quedo en el hospital porque necesito el sueldo, la remuneración para sobrevivir.* (E30)

Además, algunos ST marcan la relación de distanciamiento de las trabajadoras con sus entidades representativas de la categoría de enfermería, ya que consideran los pagos a estas organizaciones como una carga sin el rendimiento deseado.

*La profesión está abandonada, trabajando en condiciones que no son las ideales porque necesitamos dinero para sobrevivir. No tenemos representación. ¡Para afiliarse al sindicato hay que pagar! Las cuotas cobradas suponen una carga para el presupuesto y, además, tienen un bajo rendimiento y están alejadas de la profesión. Esto desmotiva.* (E12)

¡No lo veo posible! No he visto el rendimiento que esperaba por parte *del sindicato. Pagué durante años y, hasta hoy, no he visto ningún beneficio. Nada ha cambiado. Las entidades representativas de la clase están muy alejadas de la categoría. No conocen nuestros problemas y lo que necesitamos.* (E08)

*Veo a muchos colegas diciendo que el COREN^1^ y el sindicato no están haciendo nada. Solo quieren pagos cada año y no hacen nada. Ese es el discurso de nuestra clase.* (E18)

## DISCUSIÓN

En el análisis de similitud, el tamaño de las palabras y el grosor de los trazos que las unen representan la importancia de los términos para la comprensión del fenómeno estudiado^(15,16)^. En este sentido, bajo la perspectiva de dos ejes semánticos, se evidenció que, en el primero, “política” es una palabra central rodeada periféricamente por “participación política”, “deber”, “derecho”, “involucrar”, “sociedad”, “salud” y otros términos. En el segundo, el léxico “trabajo” apareció como central y se relaciona con los siguientes términos periféricos: “carga horaria”, “remuneración”, “acontecer”, “coren”, “sindicato”, “clase”, entre otros.

En esta perspectiva analítica, los léxicos centrales “política” y “trabajo” son las palabras que tienen las líneas de conexión más gruesas entre sí, lo que indica una mayor coocurrencia (aparición conjunta de estas palabras), como cuando las enfermeras relatan que la política está presente en el día a día laboral de la enfermería. Al mismo tiempo, existe un distanciamiento entre estas palabras centrales, lo que revela que la política aún no es un elemento que forme parte de las acciones intencionales diarias de la enfermería, dado que los ST muestran actitudes de evasión y distanciamiento ante temas relacionados con la política, viéndola como una tarea más en un escenario de sobrecargas y largas jornadas en el entorno laboral.

Además, los ST llevan a la comprensión de que el trabajo es solo un medio de subsistencia en la modernidad líquida y, considerando los significados del trabajo descritos por Bauman, se enfrentan dos realidades opuestas. La primera, denominada modernidad sólida, se caracterizaba por un orden social derivado de la alianza entre los Estados-nación y la ciencia. En este escenario, los trabajadores tenían poca movilidad y escolaridad, pero las relaciones con el trabajo eran duraderas y formales, siendo común ingresar al mercado laboral y jubilarse en el mismo vínculo o función. Además, el trabajo proporcionaba subsistencia económica, lo que añadía *estatus* social, seguridad, satisfacción,relaciones interpersonales y otros elementos esenciales para el ser humano y la constitución de la identidad^(4,5,6, 17, 18)^.

La segunda realidad, identificada entre las participantes de este estudio, surgió a partir de cambios derivados de crisis que transformaron la coyuntura social, política y económica, como las tragedias nucleares, los campos de concentración y el Holocausto, que derrumbaron las estructuras de la modernidad sólida. Como efecto, se inauguró la modernidad líquida como una lógica que marca las diferencias y difunde ideales de individualización, libertad, eliminación de la sociabilidad y dependencia, oponiéndose así al paradigma de la modernidad sólida que la precedió^(4,5,6, 18)^.

Esta nueva modernidad surgió de las ideas de fluidez, ligereza, precariedad, incertidumbre e inconstancia, trayendo consigo las nociones de practicidad, adaptabilidad y superficialidad de las relaciones, además de la aceleración del ritmo de vida y la exacernación de la libertad, el individualismo, el consumismo y la flexibilidad. Al mismo tiempo, se observa la expansión del capitalismo neoliberal y el debilitamiento de la relación entre el capital, el trabajo y el Estado, con el fin de favorecer la reproducción del capital de forma autónoma, globalizada y libre. En consecuencia, el Estado se ha vuelto mínimo y el trabajo flexible y precario^(4,5,6, 18)^.

En el mundo laboral, estas cuestiones se traducen en la búsqueda de trabajadores especializados y multifuncionales con relaciones laborales precarias e inestables, de modo que permanecer mucho tiempo en el mismo empleo o función es difícil y, cuando ocurre, no es bien visto, ya que refleja el estancamiento y la falta de progresión en el mercado. Ante esto, el empleo informal se expande y la identidad profesional se vuelve pasajera, reconstruyéndose constantemente a partir de estímulos relacionados con el consumo ^(4,5,6, 19)^.

Considerando que la persona es lo que consume o posee, y que el consumo ha pasado a ocupar el lugar que antes ocupaban el trabajo y las relaciones interpersonales, se tiene que, en la modernidad líquida, la identidad y las subjetividades del individuo adquieren un carácter fluido^(16,17,18,19)^. En este sentido, se comprende por qué el sentido del trabajo para las enfermeras entrevistadas se centra en las ganancias monetarias, que garantizan la subsistencia y el consumo, de modo que involucrarse en cuestiones políticas en pro de ganancias colectivas no es una prioridad. En consecuencia, la pérdida de sentido del trabajo está alineada con el vaciamiento de significados, debido a las elevadas exigencias modernas líquidas y al consumo^(19)^.

En el ámbito de la enfermería, este sombrío contexto laboral se ve agravado por las características sociodemográficas de la categoría, además de cuestiones de género, origen histórico y herencia cultural de la profesión, que conviven con la sobrecarga de tareas en el turno de trabajo, jornadas laborales extensas, salarios bajos y la desvalorización social y profesional^(4,5,6)^. En la actualidad, esta coyuntura conduce a las enfermeras a la apatía política, como se evidencia en esta investigación, a través de las subjetividades relacionadas con el trabajo, dada la prioridad del consumo, que puede desencadenar la fluidez de la identidad de estas trabajadoras.

Como corroboración, destaca la opción de las participantes por alejarse de los espacios de discusión y lucha política, a pesar de reconocer la importancia de esta participación para lograr mejoras en las condiciones laborales. De hecho, este resultado revela el individualismo, donde las aspiraciones personales están por encima de las colectivas, y el inmediatismo, que debilita la conciencia de clase como elementos naturalizados por la modernidad líquida y presentes en todas las esferas de la vida, incluido el trabajo^(4,5,6, 16,17,18,19)^.

Teniendo en cuenta el extremo del segundo núcleo de significado presentado en el árbol de similitudes, se observa que el léxico “coren” está cerca de “clase” y “sindicato”, lo que indica la correlación entre estas entidades representativas de la profesión y la categoría. Estas palabras pertenecen al rango semántico del “trabajo”, pero están periféricamente distantes, presentando una línea de conexión frágil con el término central. Así, la distancia entre estos léxicos marca que no hay una coocurrencia significativa.

Del mismo modo, los ST revelan que, en opinión de las enfermeras, las entidades representativas de la enfermería no se apropian de las necesidades reales de la categoría por no tener relaciones estrechas con las trabajadoras que la componen. Así, consideran que los valores exigidos por estas organizaciones son una barrera de acercamiento y que estos gastos impactan en el presupuesto. Por el contrario, existe la percepción de que la afiliación, la inscripción y la asociación se traducen en un bajo o nulo rendimiento en términos de beneficios colectivos para los profesionales derivados de las luchas políticas. De este modo, se conforma un ciclo de alejamiento y sensación de falta de representatividad, que va acompañado del debilitamiento de la entidad de clase y del consiguiente aumento del distanciamiento.

Desde otra perspectiva, se considera que las entidades representativas de la enfermería representan el espacio público, que no se valora en la modernidad líquida, dado que las personas se ven impulsadas a mantenerse ocupadas y preocupadas, cada vez más, con sus propias esferas privadas. En este sentido, mediante la difusión del individualismo, el yo ciudadano es sustituido por el yo individuo, de modo que, cuando el espacio público está ocupado únicamente por el yo individuo, el objetivo colectivo acaba desviándose hacia un propósito individual. Así, los pensamientos y las acciones colectivas dejan de existir, y se producen iniciativas de publicidad de la vida privada, lo que lleva al grupo a la alienación^(4,5,6, 19, 20)^.

A este contexto se suman la superficialidad de las relaciones con los pares, que impide la construcción de interacciones profundas y empáticas, y la centralidad del consumo, como componente esencial de la identidad de la persona que, al reducir la noción de ciudadanía, coloca el “ser consumidor” por encima del “ser humano”. Además, la falta de relaciones sociales genuinas y el intercambio entre la vida pública y privada, asociados al egocentrismo y la insensibilidad, impulsados por la modernidad líquida^(4,5,6, 19, 20)^, forman un panorama que aleja a los enfermeros de la esfera política y, en consecuencia, de la participación en este espacio y de la posibilidad de establecer una relación cercana y verdadera con las entidades representativas de la profesión.

Asimismo, cabe destacar la importancia de que los profesionales comprendan las competencias legales distintas de cada una de estas organizaciones, para que busquen información y ayuda a través de los canales de comunicación adecuados a la naturaleza de sus demandas^(21)^. Para ello, es necesario que las trabajadoras de enfermería rompan con la alienación impuesta por el trabajo en la era de la modernidad líquida y se acerquen a sus entidades representativas mediante la afiliación, la participación en asambleas y eventos científicos, culturales, sociales y políticos, además de la elaboración de dictámenes y el intercambio de opiniones individuales sobre temas publicados en los medios de comunicación. Estas estrategias permiten el ejercicio del control social, acercan a los profesionales a sus pares y fortalecen las instituciones propias de la profesión.

Estas cuestiones son fundamentales, ya que, en la actualidad, muchas luchas en el ámbito laboral culminan en disputas judiciales, que se enfrentan a la burocratización y la lentitud del sistema judicial. Además, estos procesos se basan en legislaciones que tienden a favorecer al capital en detrimento del trabajador. Es el caso, por ejemplo, de las reformas laborales y de la seguridad social, llevadas a cabo en los años 2017 y 2019, que, a pesar de traer consigo el discurso de la ampliación del empleo y la flexibilización de las jornadas laborales, en realidad se configuraron como movimientos de retirada de derechos de la clase trabajadora^(21,22,23)^.

En este contexto, es urgente que los profesionales de la enfermería y las entidades representativas de la profesión se organicen como un colectivo para que sus luchas conquisten espacios de poder y decisión potentes para impulsar cambios concretos en la realidad laboral de las enfermeras en medio de las relaciones de fuerza de la política, la economía y el trabajo en la modernidad líquida.

Se entiende que la limitación del estudio radica en su alcance, ya que se llevó a cabo en una sola institución de educación superior del estado de Río de Janeiro, que tiene especificidades culturales, políticas y del propio campo de la salud que no se presentan necesariamente en otros estados brasileños. Por lo tanto, los resultados pueden no ser generalizables, pero se alinean con los hallazgos de investigaciones internacionales que muestran la baja implicación de la enfermería en temas relacionados con la política^(24,25)^.

Además, este estudio ofrece bases para reflexiones críticas sobre las repercusiones de la modernidad líquida en la configuración laboral, la enfermería como colectivo profesional y las actitudes individuales en relación con las luchas colectivas en el campo político. Cabe destacar que esta problematización debe tener lugar en los espacios colectivos del trabajo, pero no se limita a los trabajadores. Es urgente que este debate impregne los procesos de enseñanza y aprendizaje desde la licenciatura hasta el posgrado, con el fin de ofrecer una formación contextualizada con el trabajo y, en consecuencia, la formación de profesionales y especialistas con competencias y disposiciones políticas para reconocer la relevancia de las entidades representativas de la categoría y actuar, como grupo, en pro de mejoras para la profesión.

## CONCLUSIÓN

Los significados que las enfermeras atribuyen al trabajo y a la política en la era de la modernidad líquida revelan que el primero es visto como un medio de subsistencia, es decir, no moviliza las subjetividades de estas trabajadoras y, por lo tanto, hay un vaciamiento de significados relacionados con el trabajo. Al mismo tiempo, reconocen que la política es importante para alcanzar beneficios colectivos, pero no muestran disposición para actuar en esta perspectiva, ya que la consideran una tarea más en el contexto de la intensificación del trabajo, al igual que ven la afiliación a las entidades representativas de la profesión como una inversión con poco o ningún rendimiento concreto.

Estos hallazgos revelan que las enfermeras se ven afectadas por el individualismo, el inmediatismo y la superficialidad como elementos naturalizados en la sociedad contemporánea que repercuten en los entornos laborales, debilitando la categoría y sus organizaciones. Ante esto, se considera que estas profesionales han incorporado los preceptos del capitalismo neoliberal inaugurado por la modernidad líquida, ya que: no se realizan en el trabajo, ya que sus actividades se limitan a recibir un salario y, por lo tanto, no producen subjetividades; no desarrollan el sentimiento de pertenencia a la clase, lo que interfiere en la construcción de la identidad; y se distancian de la implicación política, dada la fluidez de las relaciones y la desvalorización de las luchas colectivas.

## DISPONIBILIDAD DE DATOS

Todo el conjunto de datos que apoya los resultados de este estudio fue publicado en el propio artículo.

## Footnotes

1 NdT: COREN se refiere al Consejo Regional de Enfermería, un organismo de profesionales (llamado organización *de clase* en Brasil) creado por ley, de afiliación obligatoria de todo el personal de enfermería
